# Identification of Polysaccharides From *Dipsacus asperoides* and Their Effects on Osteoblast Proliferation and Differentiation in a High-Glucose Environment

**DOI:** 10.3389/fphar.2022.851956

**Published:** 2022-03-24

**Authors:** Duoduo Xu, Jia Liu, Wei Zheng, Qipin Gao, Yang Gao, Xiangyang Leng

**Affiliations:** ^1^ Country School of Pharmacy, Changchun University of Chinese Medicine, Changchun, China; ^2^ The Affiliated Hospital, Changchun University of Chinese Medicine, Changchun, China; ^3^ Jilin Ginseng Academy, Changchun University of Chinese Medicine, Changchun, China

**Keywords:** *Dipsacus asperoides* polysaccharide, extraction and separation, structure identification, osteoblast, proliferation and differentiation

## Abstract

Polysaccharides (DAI-1 and DAI-2) from *Dipsacus asperoides (D. asperoides)* were obtained using mixed-bed ion exchange resin and Sephadex G-50 column chromatography following which their properties, structures, and activities were investigated. The results showed that DAI-1 and DAI-2 were homogeneous in nature, with glucose the only constituent, and had molecular masses of 17 and 4 kDa, respectively. Methylation analysis indicated that the backbones of DAI-1 and DAI-2 were mainly composed of (1→6)-linked glucose residues. DAI-1 possessed a small number of side chains and a branch point of (1→3, 6)-glucose, while DAI-2 lacked branching*.* Activity assays demonstrated that exposing osteoblasts to different DAI-1 concentrations (25, 50, or 100 μg/mL) in a high-glucose environment induced cell proliferation and led to a significant increase in bone morphogenetic protein 2 (BMP-2) and runt-related transcription factor 2 (Runx2) expressions at both the mRNA and protein levels. Moreover, DAI-1 treatment significantly increased alkaline phosphatase (ALP) and osteocalcin (OCN) activities in osteoblasts. Combined, our results suggested that DAI-1 may promote osteoblast proliferation and differentiation in a high-glucose environment.

## Introduction

Osteoporosis (OP) is a chronic systemic metabolic bone disease characterized by bone loss, degeneration of bone microstructure, and increased bone fragility. The associated symptoms include bone pain, brittle bone-associated fractures, and paralysis. Although OP mainly affects the health of middle-aged and older adults, postmenopausal women and people with diabetes are also often prone to this condition (Compston et al., 2019).

Recent evidence has indicated that osteoblast activity is a key factor influencing OP development. Osteoblasts differentiate into mature osteoblasts in a three-stage process that involves proliferation, differentiation, and mineralization. Differentiation is required for osteoblast maturation, which is important for both bone formation and osteoblast function (Cicuéndez et al., 2017). Bone morphogenetic protein 2 (BMP-2) plays a key role in bone formation and osteogenic differentiation. Specifically, the BMP-2 signaling pathway stimulates the synthesis and secretion of bone extracellular matrix. In addition, BMP-2 induces osteoblast differentiation by activating the Smad signaling pathway and regulating osteoblast-related gene expression. Runt-related transcription factor 2 (Runx2) is another key transcription factor for osteogenic gene expression and osteoblast differentiation, and greatly influences skeletal maturity and turnover. Runx2 serves as a necessary BMP-2 osteogenesis target gene, binding to specific sequences within the promoter regions of osteoblast-specific genes and regulating their expression (Lecanda et al., 1997; Gilbert et al., 2002). Furthermore, Runx2 promotes the expression of osteoblast-specific genes, including osteocalcin (OCN) and alkaline phosphatase (ALP) (Johnson et al., 2000; Pujari-Palmer et al., 2016). Diabetes is a predisposing factor for the development of OP. Intriguingly, numerous studies on the effects of hyperglycemia on bone health have shown that this condition can inhibit osteoblast activity to some extent, not only by suppressing osteoblast proliferation and differentiation, but also by downregulating the expression of osteogenesis-related genes and proteins (Zhen et al., 2010).

Polysaccharides are natural macromolecular compounds present in the cell membranes of higher plants and animals, as well as in the cell walls of microorganisms (Zhao et al., 2017). Until recently, polysaccharides have largely been thought of as mere energy storage molecules, structural materials, or impurities for removal. However, research attention has increasingly focused on polysaccharides, as they have recently been shown to participate in a wide range of processes, including cell and molecular recognition, fertilization, growth, inflammation, autoimmunity, and the malignant transformation of tumor cells. Owing to their low toxicity, polysaccharides are currently utilized as ingredients of natural drugs and numerous health care products (Wasser, 2002; Hou et al., 2021).

*D. asperoides* is a commonly administered Chinese herbal medicine composed primarily of saponins, alkaloids, flavonoids, volatile oils, and polysaccharides (Li et al., 2017). *D. asperoides* is often used in China to treat OP and other bone diseases, and has achieved good clinical effects. However, the active components underlying its bone health-promoting effects are unknown, and studies investigating the effects of *D. asperoides* on OP have mainly focused on its saponin contents. *D. asperoides* polysaccharides have been reported to exert a range of health-promoting effects, including enhanced immunity and anti-tumor activities (Zhang et al., 1997; Cong et al., 2013; Park et al., 2019; Sun et al., 2019) and there is little research on OP. In this study, we first extracted and isolated *D. asperoides* polysaccharides and characterized their structures. Then, we investigated the effects of the isolated polysaccharides on osteoblast damage induced by a high-glucose environment. Our results lay a foundation for future studies exploring the beneficial activities of *D. asperoides* polysaccharides on human health.

## Materials and Methods

### Materials and Chemicals

*D. asperoides* samples were obtained from Jishen Pharmacy (Jilin, China) and were identified by Professor Gao as dry roots of *Dipsacus asper* Wall. ex Henry. T-series dextran standards and those of nine monosaccharides were obtained from Fluka Chemical Co. (Everett, WA, United States). Sephadex G-50 was purchased from GE Healthcare Ltd. (Chalfront St., Guiles, UK). Ion exchange resins (717 and 732) were purchased from Tianjin Xijinna Environmental Protection Material Technology Co., Ltd. (Tianjin, China).

### Isolation and Purification of *D. asperoides* Polysaccharides

**FIGURE 1:**
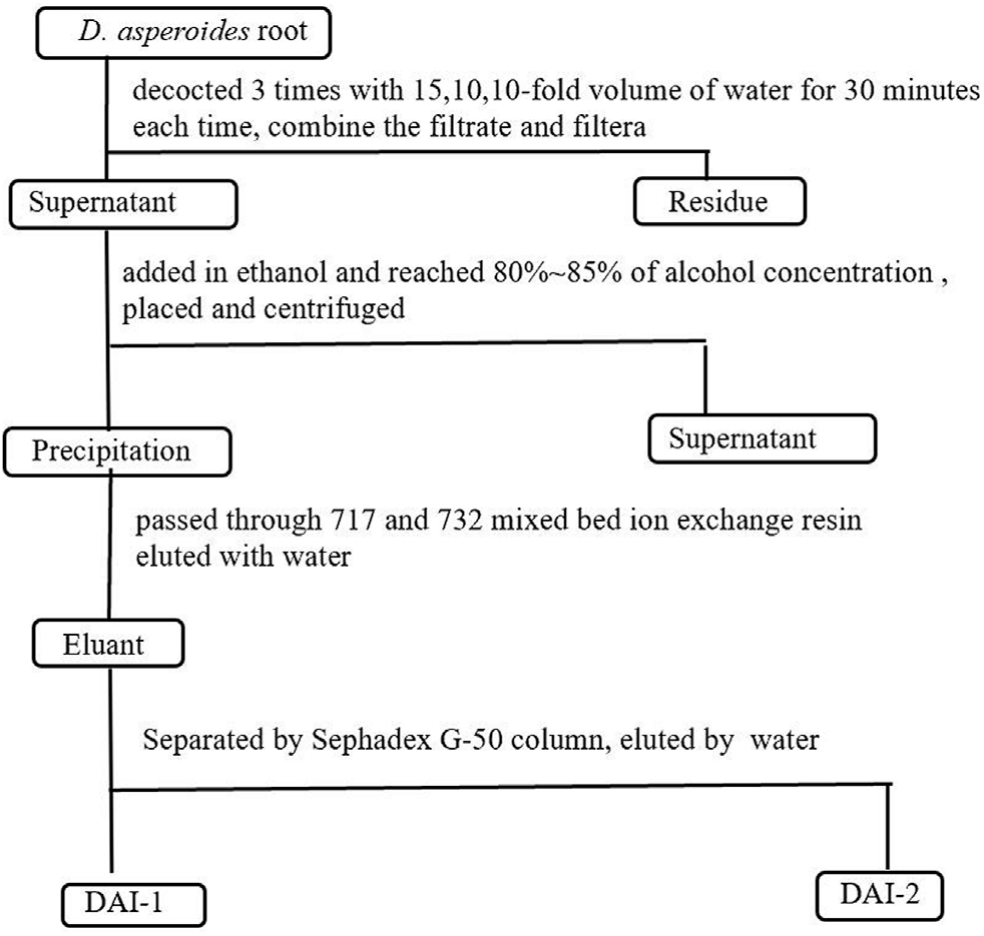
Schematic of the process used for the extraction and isolation of *D. asperoides* polysaccharides.

*D. asperoides* root material (500 g) was weighed, crushed, and soaked in a 10-fold volume of water for 2 h. For the first extraction, a 15-fold volume of water (based on the original total volume) was added, following which the mixture was decocted for 30 min, filtered, and the filtrate was retained. For the second and third extractions, a 10-fold volume (based on the original total volume) of water was added each time before decocting the mixture. The decocted mixtures were filtered and the filtrates from each extraction were retained. The three filtrates were combined and concentrated. A 95% ethanol solution was slowly added to the concentrated solution with gentle stirring until the alcohol concentration reached 80–85%. After allowing to stand for 24 h, the mixture was centrifuged and a pellet containing the precipitate was retained and dried. Next, the Sevage method (Navarini et al., 1999) was repeatedly employed to remove protein from the precipitate to yield a crude polysaccharide (DA) preparation*.* After dissolving in water, this crude polysaccharide preparation was passed through 717 and 732 mixed-bed ion exchange resin, and the bound material was eluted with water. Eluants were collected, pooled, and concentrated to generate a polysaccharide preparation designated as DAI. This preparation was further separated and purified using medium-pressure preparative liquid chromatography (CHEETAH MP, Agela, Tianjin, China) with a Sephadex G-50 column (5 × 50 cm), followed by elution using distilled water. The main DAI-1 and DAI-2 fractions were generated from pooled fractions, after which pooled DAI-1 and DAI-2 fractions were concentrated and lyophilized. The above experimental process is shown in Figure 1.

### Assays of Neutral Sugar, Uronic Acid, and Protein Contents

The neutral sugar contents of DAI-1 and DAI-2 were measured using a phenol–sulfuric acid method (Dubois et al., 2002). Uronic acid content was determined using the *m*-hydroxydiphenyl method (Blumenkrantz and Asboe-Hansen, 1973). Protein analysis was performed using the Bradford method (Bradford, 1976).

### Monosaccharide Composition Analysis

High-performance liquid chromatography (HPLC) combined with pre-column derivatization was used to determine the sugar composition of DAI-1 and DAI-2. DAI-1 (2 mg), DAI-2 (2 mg), and monosaccharide standard (1 mg) were separately derivatized according to a published method (Xu D. et al., 2016). After derivatization, the sample was dissolved in methanol, and 10 µL of the solution was injected into an Agilent 1200 HPLC system (CA, United States) equipped with a C_18_ column (4.6 µm × 250 mm). Gradient elution was performed using potassium phosphate (0.025 mol/L, pH 6.8) and acetonitrile as the mobile phase. Detection was conducted at a wavelength of 250 nm and a chromatogram was generated.

### Molecular Mass Analysis

The molecular mass of DAI-1 and DAI-2 was determined using an HPLC system (Shimadzu, Tokyo, Japan) equipped with a refractive index detector fitted with a Sepax SRT SEC-100 (7.8 × 300 mm, DE, United States) chromatographic column. For the mobile phase, 0.7% Na_2_SO_4_ was used. GPC software (China) was used to calculate the molecular mass based on the calibration curve for dextran of different molecular masses.

### Methylation Analysis

Samples were fully methylated according to the modified Ciucanu method (Needs and Selvendran, 1993) and then analyzed by Fourier transform infrared spectroscopy(IR, PerkinElmer Co., Ltd., Waltham, MA, United States) to assess methylation completion. The methylated product further acetylated to generate partially methylated and partially acetylated products that were analyzed using a gas chromatography-mass spectrometry (GC–MS) system (Agilent 6890 N/5975B) equipped with a DB-1 capillary column (30 m × 0.25 mm). The temperatures of the injection port and ion source were 200 and 250°C, respectively. The programmed heating method was as previously described (Xu D. et al., 2016). The results were analyzed based on ion flow and mass spectrographic results.

### IR Spectroscopy

Powdered polysaccharide samples were compressed with KBr to generate a solid tablet that was analyzed *via* IR Spectroscopy within the wavelength range of 400–4000 cm^−1^.

### Effects of DAI-1and DAI-2 on Proliferation and Differentiation of Osteoblasts in High Glucose Environment

#### Cell Culture

The MC3T3-E1 cell line, which is derived from mouse embryonic osteoblast precursor cells, was purchased from ATCC (Rockefeller, MD, United States). The cells were cultured in Gibco Dulbecco’s modified Eagle’s medium (DMEM) containing 10% fetal bovine serum (Thermo, Rockford, IL, United States), penicillin (105 U/mL), and streptomycin (100 mg/L) (Sigma-Aldrich, St. Louis, MO, United States) in a humidified incubator with 5% CO_2_ at 37°C. The culture medium was renewed every 2–3 days.

#### Determination of the Rates of Glucose-Mediated Inhibition of Osteoblast Proliferation Using a CCK-8 Assay

Cells were diluted to 1 × 10^5^/mL in DMEM and 100 µL of the suspension was added per well of a 96-well culture plate. Once cells had completely adhered to the wells, glucose solutions of different concentrations in serum-free medium were added to the wells followed by culture for 24, 48, and 72 h. Cells without glucose treatment served as controls. After discarding the culture medium, 10 µL of CCK-8 solution was added to each well and the plate was then incubated for 1 h. After the addition of DMSO, the optical density (OD) was measured for each well using microplate reader (Thermo, Rockford, IL, United States) at 450 nm. The absorbance values were used to calculate the rates of glucose-induced inhibition of cell proliferation.

#### Effects of DAI-1 and DAI-2 on MC3T3-E1 Cell Viability

MC3T3-E1 cells (100 µL) in the logarithmic phase were seeded in a 96-well plate at a density of 1 × 10^5^ cells/mL. Once cells had adhered to the wells, the plate was divided into a control group (DMEM), a glucose model group (75 mmol/L glucose), and three polysaccharide treatment groups (75 mmol/L glucose +25, 50, or 100 μg/mL polysaccharide). Cell viability was measured *via* CCK-8 assay after 48 and 72 h of culture.

#### Determination of ALP and OCN Activities by ELISA

Cells were inoculated into a 6-well plate and cultured for 48 h, following which the culture medium was discarded, 100 µL 0.2% Triton X-100 was added to each well, the supernatants were collected after centrifugation, and protein concentrations were determined using the BCA method. ALP and OCN activities were subsequently determined using a kit according to the manufacturer’s instructions. After color development, the OD was measured at 405 and 450 nm using a microplate reader.

#### Determination of BMP-2 and Runx2 Levels by Western Blotting

Cells (5 × 10^6^ per well) from each group were incubated in separate culture bottles. After 48 h of incubation, the cells were harvested, total protein was extracted, and protein concentrations were measured using a BCA kit (Takara Bio Inc., Shiga, Japan). Next, equal amounts (80 µg) of boiled protein were separated by 4–20% sodium dodecyl sulfate–polyacrylamide gel electrophoresis (SDS–PAGE) using GAPDH as the internal loading reference and then transferred to a polyvinylidene fluoride (PVDF) membrane (Schleicher and Schuell, Keene, NH, United States). After blocking in 5% skimmed milk for 2 h at room temperature, the membrane was incubated with primary antibody (anti-BMP-2, anti-Runx2, or anti-GAPDH, diluted 1:2000) (Proteintech Co., Manchester, UK) at 4°C overnight, washed, and then incubated with horseradish peroxidase (HRP)-labeled goat anti-mouse IgG or goat anti-rabbit IgG (1:2,000, Santa Cruz Biotechnology, Inc., Dallas, TX, United States). Band intensities were measured after detection using enhanced chemiluminescence (ECL) reagent (Thermo).

#### Quantitative PCR Assays for BMP-2 and Runx2 mRNA Expression Levels

Osteoblasts were inoculated in 12-well plates at 1 × 10^5^ cells/well and, after 2 days of polysaccharide treatment, total RNA was extracted from the five groups of osteoblasts. First-strand cDNA was synthesized from 1 μg of total RNA and was subsequently used for real-time fluorescent quantitative PCR. The mRNA expression levels of BMP-2 and Runx2 were detected using an ABI 7300 Real-time PCR System (Thermo). The primers used to amplify the BMP-2 and Runx2 sequences were synthesized by Chongqing Eternal Biotechnology Co., Ltd. and are listed in Table 1. The cycling conditions were as follows: denaturation at 95°C for 30 s, followed by 40 cycles of denaturation at 95°C for 5 s, annealing at 60°C for 30–34 s, and extension at 95°C for 15 s. Rq and Ct values were used for statistical analysis.

**TABLE 1:** The base of primers for quantitative real-time RT-PCR.

Genes	Forward primer (5-3)	Reverse primer (5-3)
BMP-2	CCT​GGG​CGG​CGC​GGC​CGG​CCT​CAT​T	AGC​CGG​TGG​TCT​GGG​GCG​GGC​GCT
Runx2	GGG​GCA​GTC​ATA​ACT​GGG​TT	GCG​TGG​GAA​CAG​GTC​ACT​TA
GAPDH	TTG​TGC​AGT​GCC​AGC​CTC​GTC​CCG	TGC​CAC​TGC​AAA​TGG​CAG​CCC​TGG​T

### Statistical Analysis

Data were expressed as means ± SD. Differences among groups were analyzed using one-way analysis of variance (ANOVA) with the Cochran-Armitage test; dose-effect trend analysis was conducted using SAS software. Differences were considered significant at *p* < 0.05.

## Results

### Extraction and Purification of DAI-1 and DAI-2

**FIGURE 2:**
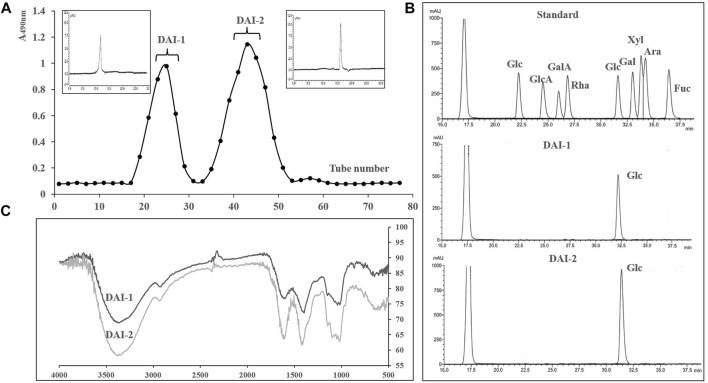
The elution curve for DAI-1 and DAI-2 passed through Sephadex G 50 columns. The upper left and upper right panels correspond to their gel permeation chromatograms **(A)**. Liquid chromatograms of DAI-1, DAI-2, and monosaccharide standards **(B)**. Infrared chromatograms of DAI-1 and DAI-2 **(C)**.

Crude polysaccharide preparations of *D. asperoides* were isolated *via* water extraction and alcohol precipitation, with a final yield of 28.3%. Determination of physical and chemical properties indicated that these extracts mainly contained neutral polysaccharides, proteins, and other water-soluble impurities, with almost no acid sugar. Accordingly, free protein and other impurities in the crude polysaccharide were removed using 717 and 732 mixed-bed ion exchange resin, and the DAI were further purified using Sephadex G-50 columns. The elution curve is shown in Figure 2A.

### Basic Properties of DAI-1 and DAI-2

The sugar contents of DAI-1 and DAI-2 were 98.3 and 99.1%, respectively, with almost no acid sugar or protein being detected. The absence of nucleic acid and protein was further confirmed by the lack of absorption peaks at 260 and 280 nm. Sugar composition analysis further showed that these polysaccharide preparations contained only glucose (Figures 2A,B). For molecular mass determination, DAI-1 and DAI-2 presented with single, sharp absorbance peaks, indicating that they were homogeneous polysaccharides and had molecular masses of 17 and 4 kDa, respectively.

### Methylation Analysis

Determination of polysaccharide structure has always been a difficult challenge in polysaccharide research. Although nuclear magnetic spectroscopy is increasingly used for the determination of polysaccharide structure, analysis of methylation remains an important method for this purpose as it can provide a large amount of information, such as details regarding the types and proportions of sugar residues. Methylation results for DAI-1 and DAI-2 were deduced by analyzing information within specific mass spectra regions, as outlined in the ion flow diagram in Table 2. Data analysis indicated that DAI-1 contains three types of glycosidic linkages in a molar ratio that reflects the presence of a few side chains, whereas DAI-2 is a linear (unbranched) polysaccharide. The numbers of ends and branches for DAI-1 were the same, thereby confirming that this result was reasonable, while (1→6) linkages between glucose residues comprised the main linkage type detected in both DAI-1 and DAI-2.

**TABLE 2:** Methylation analysis data of DAI-1 and DAI-2.

Polysaccharide	Retention time	Methylated sugar	Molar ratio	Deduced linkage pattern	Main mass spectrum fragment ion
DAI-1	20.2	2,3,4,6-Me_4_- Glc	4.9	T- Glc*p-*(1→	87,101,117,129,161,205
	24.5	2,4,6- Me_3_- Glc	40.2	→3)- Glc*p-*(1→	87,101,117,129,161,233
	30.4	2,4- Me_3_- Glc	4.9	→3,6)- Glc*p-*(1→	87,117,129,189,233,261
DAI-2	20.2	2,4,6-Me4- Glc	1.6	→3)- Glc*p*-(1→	87,101,117,129,161,233
	24.5	2,3,4- Me3- Glc	6.0	→6)- Glc*p*-(1→	87,101,117,129,161,189,233

Note: Glc stands for glucose.

### IR Analysis

The IR chromatograms of DAI-1 and DAI-2 were similar and contained absorption peaks characteristic of polysaccharides (Figure 2C). Taking DAI-1 IR spectra as an example, the absorbance band at 3371 cm^−1^ in the IR chromatogram is characteristic for the O-H stretching vibration associated with polysaccharides; the weak band at approximately 2927 cm^−1^ corresponds to the C-H stretching vibration of carbohydrates; the 1597 cm^−1^ band corresponds to a C-O stretching vibration; and the absorption bands at 1408 cm^−1^ were associated with C-O stretching vibration. The strong band at 1016 cm^−1^ indicated that DAI-1 possessed a pyran-type structure (Gong et al., 2017). Additional absorption bands at approximately 866 and 738 cm^−1^ indicated that the sugar units of DAI-1 assumed *ß*- and *a*-configurations, respectively (Zhao et al., 2014).

### Effects of DAI-1and DAI-2 Treatment on Osteoblast Proliferation and Differentiation

#### Determination of the Effects of Different Glucose Concentrations on Osteoblast Growth Rates

**FIGURE 3:**
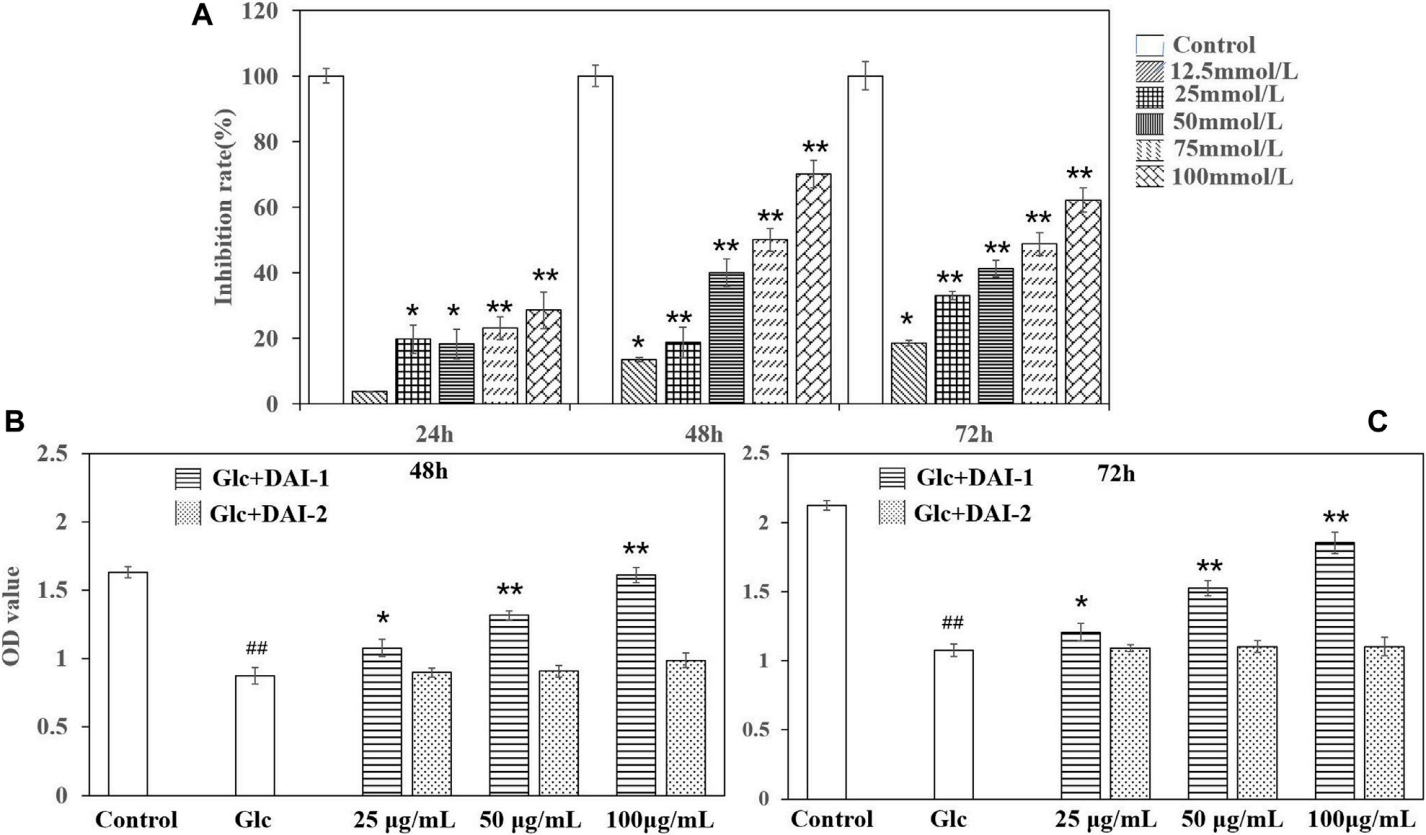
The effects of different glucose concentrations on osteoblast growth rates **(A)**. *: *p* < 0.05, **: *p* < 0.01, for high-glucose group (represented by ‘Glc’ in the figure) *vs*. the control group (represented by ‘Control’ in the figure). The effects of exposing osteoblasts to DAI-1 and DAI-2 under a high-glucose environment for 48 h **(B)** and 72 h **(C)**. ^##^: *p* < 0.01, for high-glucose group *vs*. the control group; *: *p* < 0.05, **: *p* < 0.01, for the DAI-1and DAI-2 treated group *vs*. high-glucose group.

As shown in Figure 3A, osteoblast growth was inhibited in a manner that was dependent on the glucose dose and culture duration. When glucose was administered at the concentration of 25 mmol/L for 24 h, osteoblast growth was significantly inhibited. At the concentration of 100 mmol/L, the inhibition rate reached approximately 35%. At the glucose concentrations of 50 and 75 mmol/L, the rate of osteoblast growth inhibition ranged from 40–60% after 48 h of treatment, similar to that seen at 72 h. Considering factors such as incubation time, we selected the glucose concentration of 75 mmol/L and treatment for 48 h as the modeling parameters in this experiment as this resulted in a stable osteoblast growth inhibition rate of between 40 and 60%.

#### Effects of DAI-1 and DAI-2 on the Proliferative Ability of MC3T3-E1 Cells Exposed to a High Glucose Concentration

The effects of DAI-1 and DAI-2 treatment on the proliferation of MC3T3-E1 cells after exposure to high glucose concentrations are shown in Figures 3B,C. Cells treated with low, medium and high doses of DAI-1 exhibited significantly increased cell proliferation compared with that in cells treated with glucose only, with increases of 1.23-, 1.50-, and 2.11-fold after 48 h of treatment and 1.12-, 1.42-, and 1.72-fold after 72 h of treatment, respectively, thus providing evidence of a dose-dependent effect for DAI-1. In contrast, compared with cells treated with glucose only, exposure to DAI-2 did not affect (*p* > 0.05) osteoblast proliferation in a high-glucose environment irrespective of the concentration used or duration of treatment (48 or 72 h).

#### Effects of DAI-1 and DAI-2 Treatment on ALP and OCN Activities as Determined by ELISA

**FIGURE 4:**
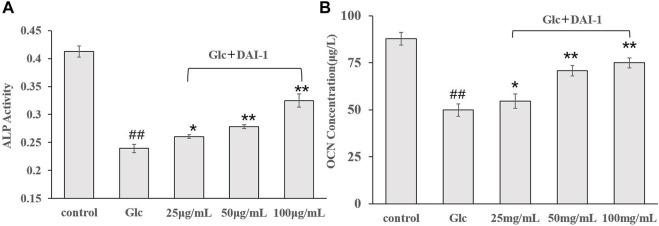
The effects of DAI-1 on alkaline phosphatase (ALP) **(A)** and osteocalcin (OCN) **(B)** in osteoblasts exposed to high glucose concentrations. Osteoblasts were cultured in different conditioned media for 48 h, following which ALP and OCN activities were determined. ^##^: *p* < 0.01, for high-glucose group *vs*. the control group; *: *p* < 0.05, **: *p* < 0.01, for the DAI-1and DAI-2 treated group *vs*. high-glucose group.

ALP activity is an important marker of early-stage osteoblast differentiation and a functional indicator of osteoblast differentiation status, while OCN is a marker of late osteoblast differentiation and a key regulator of bone mineralization. The effects of DAI-1 on ALP and OCN activities in MC3T3-E1 cells exposed to high-glucose conditions are shown in Figure 4. Compared with the control group, ALP and OCN activities in the high-glucose treatment group showed a significant decline after 48 h (ALP: 0.57-fold, *p* = 1.56 × 10^–5^; OCN: 0.57-fold, *p* = 4.31 × 10^–5^, respectively). In contrast, ALP and OCN activities increased significantly in the DAI-1-treated groups; compared with cells treated with glucose only, those exposed to glucose plus low, middle, or high DAI-1 concentrations displayed changes of 1.09- (*p* = 0.012), 1.17- (*p* = 0.00118), and 1.36-fold (*p* = 0.000459), respectively, in ALP activity and 1.08- (*p* = 0.03294), 1.42- (*p* = 0.00015), and 1.50-fold (*p* = 7.29 × 10^–5^) in OCN activity. These results indicated that DAI-1 dose-dependently promoted ALP and OCN activities in ME3T3-E1 cells in a high-glucose environment. As observed for the proliferation effect, DAI-2 did not significantly affect ALP or OC activities in osteoblasts under a high-glucose environment regardless of the concentration used (*p* > 0.05). This indicated that DAI-2 had no effect on osteoblast differentiation.

#### DAI-1 and DAI-2 Inhibited the Protein Expression of BMP-2 and Runx2 in Osteoblasts Exposed to High-Glucose Conditions

**FIGURE 5:**
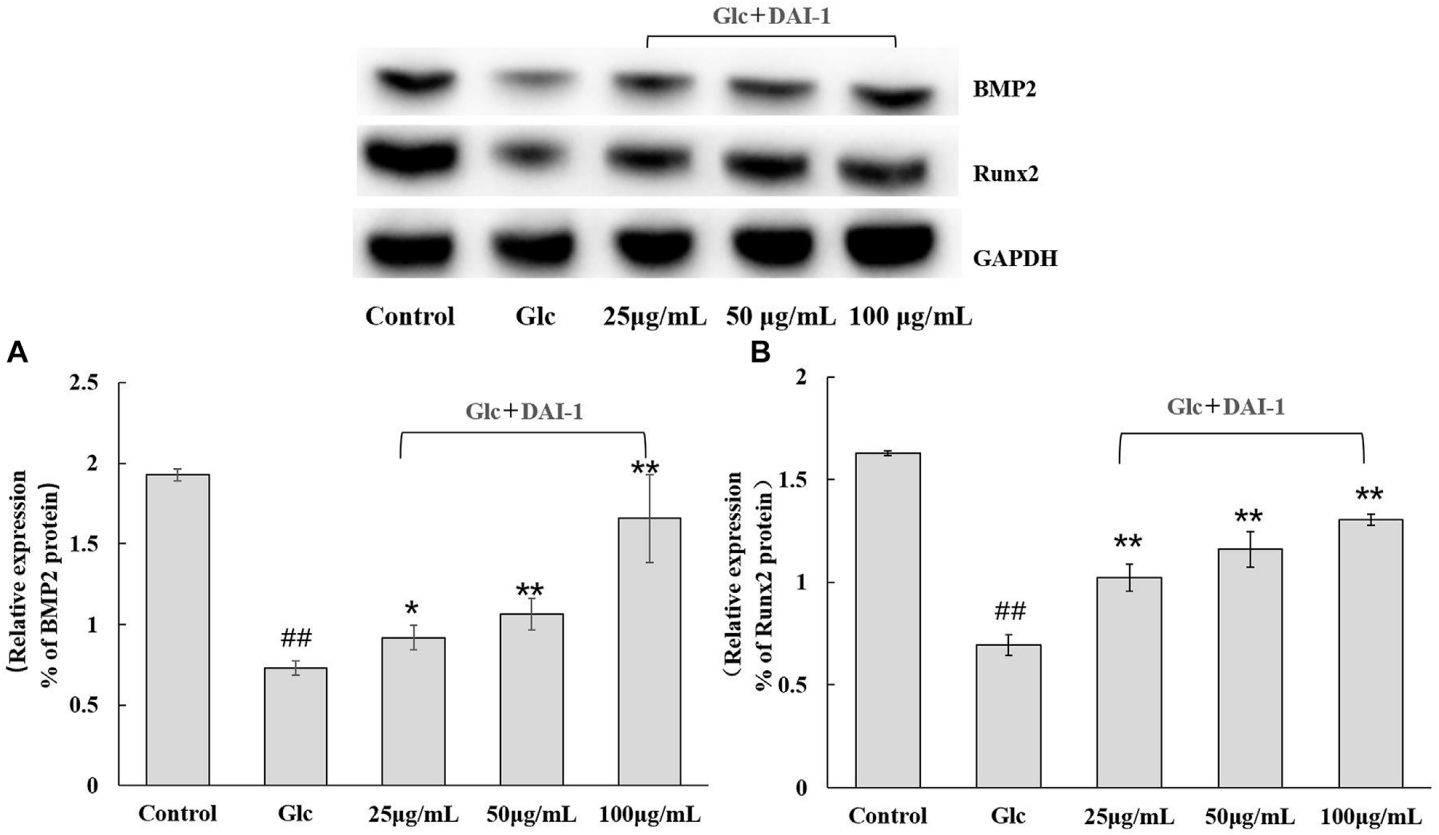
The effect of DAI-1 on the relative protein expression of the osteoblast differentiation-related factors BMP-2 **(A)** and Runx2 **(B)** in a high-glucose environment. After 48 h of treatment, the protein levels of BMP-2 and Runx2 in osteoblasts were quantified by western blot. GAPDH served as a loading control. ^##^: *p* < 0.01, for high-glucose group *vs*. the control group; *: *p* < 0.05, **: *p* < 0.01, for the DAI-1and DAI-2 treated group *vs*. high-glucose group.

The protein expression levels of BMP-2 and Runx2 in osteoblasts were monitored after 48 h of DAI-1 treatment in a high-glucose environment (Figure 5). No significant difference (*p* > 0.05) in the levels of these proteins were found between the high-glucose treatment group and the group exposed to glucose and DAI-2 (data not shown) in osteoblasts. The expression levels of BMP-2 and Runx2 in the high-glucose treatment group were significantly lower than those of the control group (0.38-fold [*p* = 3.5 × 10^–6^] and 0.42-fold [*p* = 6.0 × 10^–6^], respectively). In contrast, DAI-1 treatment upregulated the protein expression levels of BMP-2 and Runx2 [1.25- (*p* = 0.021), 1.46- (*p* = 0.0059), and 2.27-fold (*p* = 0.0043); and 1.47- (*p* = 0.0023), 1.67- (*p* = 0.0012), and 1.88-fold (*p* = 4.7 × 10^–5^), respectively, for the low, medium, and high DAI-1 concentrations]. These data demonstrated that DAI-1 treatment could markedly reverse the high-glucose-induced inhibition of BMP-2 and Runx2 protein expression.

#### DAI-1 and DAI-2 Inhibited the mRNA Expression of BMP-2 and Runx2 in Osteoblasts Under High-Glucose Conditions

**FIGURE 6:**
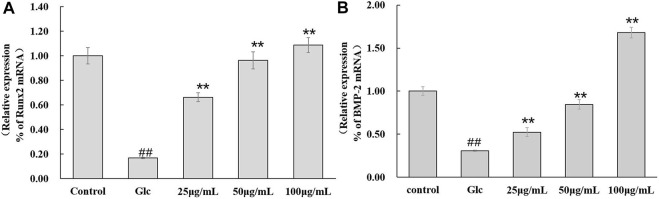
The effect of DAI-1 on the relative mRNA expression of the osteoblast differentiation-related factors Runx2 **(A)** and BMP-2 **(B)** in a high-glucose environment. After 48 h of treatment, the transcription levels of Runx2 and BMP-2 in osteoblasts were quantified using quantitative real-time PCR. ^##^: *p* < 0.01, for high-glucose group *vs*.the control group; *: *p* < 0.05, **: *p* < 0.01, for the DAI-1and DAI-2 treated group *vs*. high-glucose group.

The effect of DAI-1 treatment on BMP-2 and Runx2 mRNA levels is shown in Figure 6. After 48 h of culture, glucose treatment led to a decrease in the mRNA expression levels of BMP-2 (0.31-fold, *p* = 3.0 × 10^–5^) and Runx2 (0.17-fold, *p* = 2.3 × 10^–5^) relative to those in the control group. Compared with cells treated with glucose only, those exposed to glucose plus a high DAI-1 concentration displayed the greatest increases in BMP-2 (5.5-fold, *p* = 2.5 × 10^–5^) and Runx2 (1.1-fold, *p* = 0.0003) mRNA expression. Meanwhile, no significant difference in the mRNA expression of BMP-2 and Runx2 was observed with DAI-2 treatment (*p* > 0.05, data not shown).

## Discussion

Our findings showed that exposure to DAI-1, but not DAI-2, could promote osteoblast proliferation and differentiation in a high-glucose environment. This result was likely closely related to differences in the structure of the two polysaccharides. The molecular weight, monosaccharide composition, glycosidic bond, advanced structure and other structural characteristics of polysaccharides can affect the activity of polysaccharides (Ryoyama et al., 2014; Xiao et al., 2014; Xu F. et al., 2016). We have previously shown that the molecular mass of *Tremella* polysaccharide is the decisive factor affecting its immune activity (Gao et al., 2020). In this experiment, although DAI-1 and DAI-2 have the same composition and type of sugar residue, they differ in molecular mass and the presence/absence of branched chains. Future studies should focus on investigating the effect of polysaccharide structure on osteoblast activity.

Diabetic OP is a disorder of bone metabolism caused by long-term exposure to high-glucose conditions and has been associated with impaired osteoblast function. Osteoblasts, key regulators of bone formation, have important functions in bone matrix synthesis and secretion, bone mineralization, and bone remodeling. When osteoblast function is compromised or osteoblast numbers are reduced, the bone resorption rate exceeds the rate of bone formation, leading to decreased bone mass and, consequently, OP. Osteoblast differentiation, an important stage of bone formation, comprises three main phases, namely, cell proliferation, extracellular matrix maturation, and mineralization. Intriguingly, a high-glucose environment can repress the expression of genes and proteins related to osteogenic differentiation, resulting in the inhibition of osteoblast proliferation and differentiation.

ALP is a reliable marker of the early stage of osteoblast differentiation and serves as a functional indicator of osteoblast differentiation. ALP plays a major role in calcification *in vivo* and its positively correlated with osteoblast maturation status. Another osteoblast marker, OCN, is associated with the bone mineralization process during late osteoblast differentiation. Importantly, ALP and OCN activities undergo specific changes that are characteristic of each type of OP, and thus have greater sensitivity and specificity for use in monitoring osteoblast differentiation when compared with other osteoblast-associated markers (Song et al., 2009; Guzman et al., 2012; Runyan et al., 2012; Kopf et al., 2014). Meanwhile, the BMP-2/Smad/Runx2/Osterix signaling pathway plays an important role in osteoblast differentiation and the synthesis and secretion of extracellular matrix components. Runx2 protein regulates the expression of its corresponding gene when BMP signals are transmitted *via* the phosphorylation of Smad1/5/8, important regulators of osteoblast differentiation and bone development. As a transcription factor, Runx2 can also promote the expression of genes encoding osteoblast-specific proteins (e.g., OCN).

In this study, we isolated DAI-1 from *D. asperoides*, a traditional Chinese medicine used for tonifying kidney and strengthening bone, and characterized its structure. Under high-glucose conditions, osteoblasts (MC3T3-E1 cells) exposed to DAI-1 exhibited a significant increase in BMP-2 and Runx2 expression at both the mRNA and protein levels. Moreover, DAI-1 treatment reversed the loss of the activities of ALP and OCN resulting from exposure to a high-glucose environment. Our data suggest that DAI-1 treatment may reverse the inhibition of MC3T3-E1 cell proliferation and differentiation induced by high levels of glucose, effects that are likely mediated through the stimulation of the BMP-2/Smad/Runx2/Osterix signaling pathway.

## Data Availability

The datasets used and/or analyzed during the current study are available from the corresponding author on reasonable request.
